# Cell proliferation and apoptosis in rat mammary glands following combinational exposure to bisphenol A and genistein

**DOI:** 10.1186/1471-2407-14-379

**Published:** 2014-05-29

**Authors:** Jun Wang, Sarah Jenkins, Coral A Lamartiniere

**Affiliations:** 1 Department of Pharmacology and Toxicology, University of Alabama at Birmingham, Birmingham, AL 35294, USA; 2 UAB Comprehensive Cancer Center, University of Alabama at Birmingham, Birmingham, AL 35294, USA

**Keywords:** Bisphenol A, Genistein, Proliferation, Apoptosis, Mammary cancer

## Abstract

**Background:**

Humans are exposed to an array of both harmful and beneficial hormonally active compounds in the environment and through diet. Two such chemicals are Bisphenol A (BPA), a plasticizer, and genistein, a component of soy. Prepubertal exposure to BPA increased mammary carcinogenesis, while genistein suppressed cancer in a chemically-induced model of rodent mammary cancer. The purpose of this research was to determine the effects of combinational exposure to genistein and BPA on cell proliferation, apoptosis, and associated proteins as markers of cancer in mammary glands of rats exposed prepubertally to these environmental chemicals.

**Methods:**

Prepubertal rats (postpartum days (PND) 2–20) were exposed through lactation via nursing dams treated orally with sesame oil (SO), BPA, genistein, or a combination of BPA and genistein (BPA + Gen). Cell proliferation, apoptosis and protein expressions were investigated for mechanistic studies in mammary glands of rats exposed to these environmental chemicals.

**Results:**

Prepubertal exposure to genistein increased cell proliferation in mammary glands of PND21 rats, while BPA increased cell proliferation in adult (PND50) rats. Prepubertal combinational exposure to BPA + Gen increased cell proliferation and reduced apoptosis in PND21 rats, but reduced cell proliferation and increased apoptosis in PND50 rats. The altered mechanisms behind these cellular responses appear to be centered on differential protein expression of caspases, PARP, Bad, p21, Akts, PTEN, ER-β and SRCs 1–3, in the rat mammary gland.

**Conclusion:**

Prepubertal BPA exposure resulted in increased cell proliferation in mammary glands of PND50 rats, a process associated with increased risk of cancer development in a chemically-induced mammary cancer. On the other hand, genistein stimulated cell proliferation at PND21, a process that correlates with mammary gland maturation and chemoprevention. In contrast to single chemical exposure, combinational exposure to BPA + Gen performed most similarly to genistein exposure alone. BPA + Gen increased cell proliferation at PND21, suggesting mammary gland maturation, and decreased cell proliferation while increasing apoptosis in PND50 rats, suggesting mammary chemoprevention. Differential expression of proteins involved in regulating cell proliferation and apoptosis lend support to these chemicals, both alone and in combination, altering mammary gland cancer susceptibility.

## Background

Epidemiological studies suggest that exposure to environmental estrogens affects cancer rates [1]. However, the exact effects of environmental estrogens on the biology of the mammary gland remain elusive. Such is the case of the plasticizer, BPA, and the isoflavone, genistein. BPA is one of the highest volume chemicals produced worldwide, with an estimated 7% annual growth due to manufacturing demand. It is used as a monomer to manufacture polycarbonate plastics, epoxy resins that line most food and beverage cans, and dental sealants. Time, heat, and acidic or basic conditions accelerate hydrolysis of the ester bond linking BPA monomers, leading to the release of BPA into food and liquid and making human environmental exposure inevitable and ubiquitous [2]. In the United States, recent measurements by the Center for Disease Control revealed detectable levels of BPA in random spot collected urine samples from 92.6% of more than 2500 participants in the cross-sectional National Health and Nutrition Examination Survey study [3]. Further, BPA has been detected in amniotic fluid, maternal and fetal plasma, placental tissue of pregnant women, and in breast milk of lactating mothers [4,5].

Genistein is a phytoestrogen found in many food products, especially soy-based foods such as tofu, soymilk, soy infant formula and in some over-the-counter dietary supplements. Dietary exposure to genistein can reach up to 1 mg/kg body weight (BW) in adults and up to 10-fold higher levels in infants fed milk formulas containing soy [6]. The level of genistein exposure in Asian populations consuming a soy-rich diet has been reported to range from ~1 to 30 mg/d, or ~0.02–0.55 mg/kg BW × d, and considerably less in Western populations [7]. Oral exposure to genistein during the early prepubertal period decreased susceptibility of the mammary gland to carcinogenesis [8-10], whereas exposure during the same period of time to BPA increased susceptibility to chemically-induced mammary cancer [9,11,12]. Although both BPA and genistein possess weak estrogenic properties, these chemicals appear to influence the mammary gland via unknown, yet different, mechanisms.

To better understand the mechanisms of BPA and genistein exposure, both alone and in combination, we investigated the effects of prepubertal BPA and/or genistein exposure on cell proliferation and apoptosis in the structures most susceptible to mammary carcinogenesis, the terminal end buds (TEBs). Further, we assessed the expression of proteins in signalling pathways implicated in mammary gland maturation, cell proliferation, and apoptosis in the mammary glands of PND21 and PND50 rats. Differential cell proliferation, apoptosis, and protein expression patterns in the mammary glands of rats prepubertally exposed to BPA and genistein, both alone and in combination, suggest mechanisms for the altered susceptibility of the mammary gland to chemically induced carcinogensis.

## Methods

### Animals

Animal care and treatments were performed according to established guidelines, and protocols were approved by the UAB Animal Care Committee. Seven week old female Sprague–Dawley rats were purchased from Charles River Laboratories (Wilmington, MA). Animals were housed in a temperature controlled facility with a 12-h light:dark cycle. These animals were bred with proven Sprague–Dawley studs in our facilities, fed phytoestrogen-free AIN-76A diet (Harlan Teklad, Madison WS), housed in polypropylene cages and water provided via glass bottles (all polycarbonate/BPA free). On the day of birth (designated as PND zero), offspring were sexed and litters were culled to 10 offspring per lactating dam. Beginning on PND2 and continuing through PND20, the lactating dams were treated as listed below (Table 1).

**Table 1:** A list of animal treatment

**Group identification**	**Gavage administered**	**Food administered**
1). Control (SO)	Sesame Oil as Vehicle	AIN-76A
2). Bisphenol A (BPA)	250 ug BPA/kg BW	AIN-76A
3). Genistein (Gen)	Sesame Oil as Vehicle	250 mg Genistein/kg AIN-76A diet
4). BPA + Genistein (BPA + Gen)	250 ug BPA/kg BW	250 mg Genistein/kg AIN-76A diet

Genistein was provided by DSM Nutritional Products (Basel, Switzerland). BPA, sesame oil, and all other chemicals were from Sigma Chemical Co. (St. Louis, MO). Offspring were weaned at PND21 and fed AIN-76A diet. For biochemical studies, female offspring were killed on PND21 and 50 (prepubertal and young adult rats, respectively). At PND50, female offspring were killed in the estrous phase. The fourth abdominal mammary glands were rapidly dissected from live ketamine/xylazine anesthetized animals (to minimize proteolysis), snap-frozen in liquid nitrogen, and stored at -80ºC or placed in 10% formalin for later analysis.

### Immunohistochemistry

Immunohistochemistry (IHC) of tissue sections from formalin fixed mammary glands was employed to measure Ki-67 antigen as a marker for proliferating cells in mammary glands of PND21 and PND50 rats (*n* = 6 per group). After being blocked in 2.5% normal horse serum, sections were incubated with monoclonal mouse anti-rat Ki-67 antigen (clone MIB-5 antibody, Dako Cytomation, Carpinteria, CA), and diluted in PBS with 1% BSA for 30 min. Chromogen, diaminobenzidine (DAB) (Vector) was applied to samples for 10 min followed by a wash in tap water for 5 min. To counter-stain, hematoxylin QS (Vector) was applied to the specimens for one min followed by a wash in tap water. The slides were evaluated using a Nikon Labophot-2 microscope (Nikon Corporation, Tokyo, Japan), and data were digitally recorded using a Nikon 8.0 Mega Pixels CoolPix 8700 Digital Camera (Nikon). The pictures were counted using Image J software (Image J, NIH). The epithelial cells of TEBs positive for Ki-67 (stained brown) were counted as well as the non-proliferative epithelial cells (stained blue). Approximately 800 epithelial cells per specimen were counted in the TEBs of mammary glands. A TEB is characterized as an elongated ductal structure with 3 to 6 epithelial cell layers and >100 micrometers in diameter [13]. The proliferative index was calculated by dividing the number of positively stained cells by the total number of cells counted and expressed as a percentage.

### In situ apoptosis labelling

For apoptosis assessment, the TUNEL assay was performed with ApopTag Plus Peroxidase In Situ Apoptosis Detection kit (Chemicon International, Temecula, CA) following the manufacturer’s instructions. For quantitation of apoptotic cells, two parameters were used to identify apoptotic cells. First, we used positive immune-labelling as detected by ApopTag staining. Second, cells that were positive by immune-labelling were then confirmed by morphological characteristics typical of cells undergoing programmed cell death, i.e., including chromatin aggregation, nuclear, and cytoplasmic condensation as well as fragmentation of the dying cell into a cluster of membrane bound segments. If a cell was judged to be apoptotic by both criteria, it was judged positive. At PND21 and PND50, cell apoptotic indices were generated by counting over 1000 cells in the TEBs in more than four separate microscopic fields showing the highest density of immunostained cells per slide under a microscope and expressed as the percentage of positive cells per total number of TEB epithelial cells counted (*n* = 6 per treatment).

### Immunoblotting

To determine the changes in protein expression, six mammary gland samples per treatment group were investigated by western blot analysis. The same quantity of protein from each sample was separated by SDS-PAGE and transferred to a nitrocellulose membrane (Bio-Rad, Hercules, CA). The membranes were blocked and immunoblotted with appropriate antibodies. Molecular weight ladders (Bio- Rad) and reference proteins from the respective companies were used as positive controls. Nitrocellulose membranes were incubated with an appropriate secondary antibody conjugated to HRP (Cell Signaling Technology), followed with a chemiluminescent substrate (Pierce) and exposed to X-ray radiography film. Table 2 provides the identity, description, commercial source, and conditions of the primary and secondary antibodies used in immunoblotting. Quantitative analysis of protein expression was accomplished by scanning autoradiogram and densitometry (ImageJ, NIH).

**Table 2:** Primary and secondary antibodies for immunoblot analysis

**Primary Antibodies**					
**Antibody name**	**Supplier**	**Product number**	**Source**	**Dilution**	**Molecular weights**
Akt-1	Cell Signaling	2938	Rabbit	1:1000	60 kDa
Akt-2	Cell Signaling	5239	Mouse	1:1000	60 kDa
Akt-3	Cell Signaling	3788	Rabbit	1:1000	60 kDa
p-Akt	Cell Signaling	4060	Rabbit	1:2000	60 kDa
Cleaved Caspase-3	Cell Signaling	9661	Rabbit	1:1000	17/19 kDa
Cleaved Caspase-9	Cell Signaling	9507	Rabbit	1:1000	17 kDa
PARP	Cell Signaling	9542	Rabbit	1:1000	89/116 kDa
p-Bad	Cell Signaling	9296	Mouse	1:1000	23 kDa
p21	Cell Signaling	2946	Mouse	1:1000	21 kDa
PTEN	Cell Signaling	9552	Rabbit	1:1000	54 kDa
ER-α	R&D System	MAB57151	Mouse	1:2000	65 kDa
ER-β	Santa Cruz	sc-8974	Rabbit	1:200	56 kDa
SRC-1	BD Biosciences	612378	Mouse	1:250	165 kDa
SRC-2/TIF2	BD Biosciences	610984	Mouse	1:250	160 kDa
SRC-3/AIB-1	BD Biosciences	611105	Mouse	1:250	160 kDa
GAPDH	Cell Signaling	2118	Rabbit	1:1000	38 kDa
Secondary Antibodies					
Antibody Name	Supplier	Product Number	Source	Dilution	
Anti-Rabbit IgG HRP	R&D System	HAF008	Goat	1:2000	
Anti-Mouse IgG HRP	R&D System	HAF007	Goat	1:2000	

### Statistical analysis

Statistical analyses of morphometric and biochemical analyses were performed by using one way analysis of variance (ANOVA), with subsequent post-hoc comparisons using pre-planned linear contrast to determine significance (P < 0.05).

## Results

### Vaginal opening, body weights, and organ weights

Beginning at 24 days postpartum, female offspring were evaluated for day of vaginal opening, and body weight at the day of vaginal opening. Average time to vaginal opening was 33.8 ± 0.3, 33.7 ± 0.3, 33.5 ± 0.3, and 33.9 ± 0.4 days for SO (controls), genistein, BPA, and BPA + Gen treatments, respectively (p = 0.09). Average weight at time of vaginal opening was 118.7 ± 2.1, 114.1 ± 3.7, 110.8 ± 1.9, and 117.0 ± 2.6 grams for SO, genistein, BPA, and BPA + Gen treatments, respectively (p = 0.24). Body weights were assessed at PND7, 14, 21, 50, and 100 and no significant differences were observed between any of the treatment groups (data not shown). Uteri and ovaries were dissected out and weighed on PND21, 50, and 100. No significant changes were noted between any of the groups (data not shown).

### Cellular proliferation and apoptosis in the mammary glands of PND21 rats

**Figure 1:**
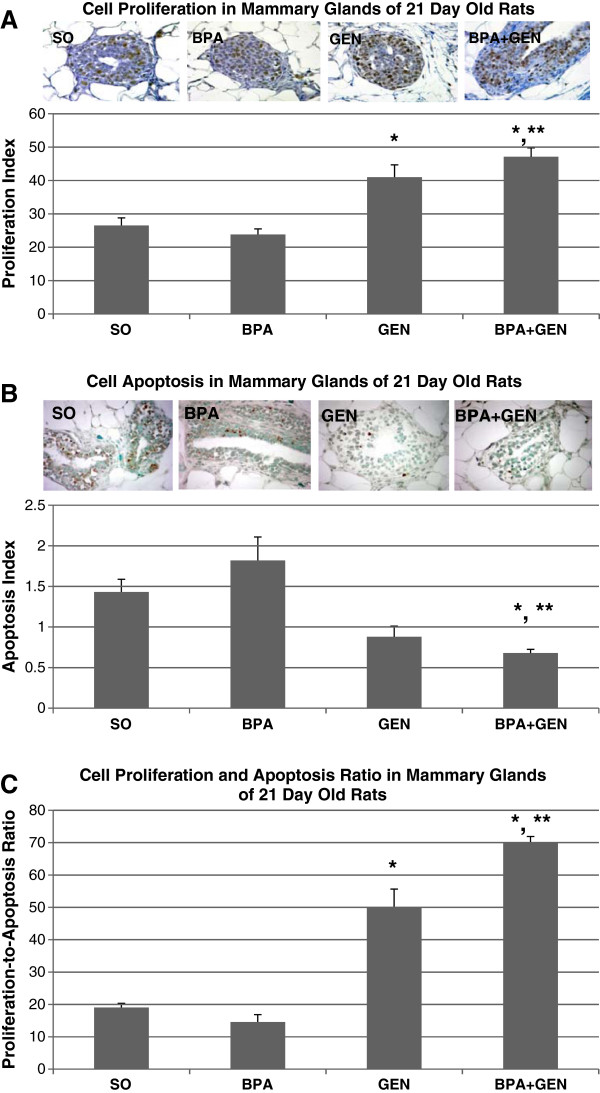
**Cell proliferation and apoptosis in PND21 rats.** Cell proliferation **(A)**, apoptosis **(B)**, and cell proliferation-to-apoptosis ratio **(C)** in mammary gland TEB epithelial cells of PND21 rats exposed prepubertally via lactating dams treated with SO (Control), or BPA, or genistein or BPA plus genistein (n = 6 rats/treatment) from PND2 through PND20. Values represent mean ± SEM. *p ≤ 0.01 compared with controls; **p ≤ 0.001 compared with BPA.

At PND21, rats exposed prepubertally to genistein exhibited a significant increase in the cell proliferation index in mammary gland TEBs (p < 0.01) as compared to controls (SO) (Figure 1A). BPA + Gen combination treatment significantly increased cell proliferation compared to control as well as BPA only exposure (p < 0.001). In the analysis of apoptosis, prepubertal BPA + Gen combination treatment reduced apoptosis in the mammary glands of PND21 rats as compared to control (p < 0.001) and BPA only exposure (Figure 1B). Using the ratio of cell proliferation and apoptosis to estimate cell turnover in mammary TEBs, genistein only exposure increased the ratio compared with controls (p < 0.01) (Figure 1C), Likewise, BPA + Gen exposure resulted in an increased cell proliferation to apoptosis ratio in mammary TEBs of PND21 rats.

### Protein expression in mammary glands of PND21 rats

**Figure 2:**
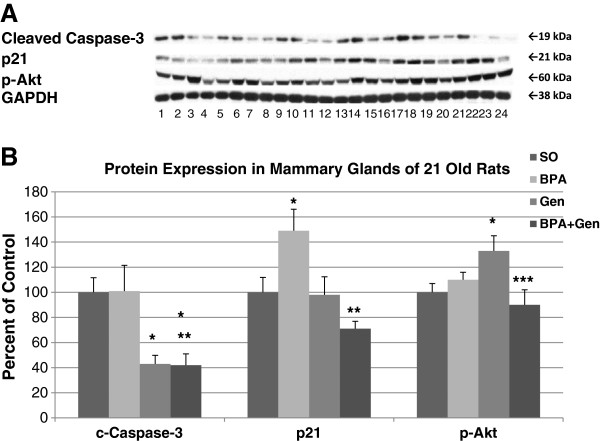
**Protein expressions in PND21 rats.** Western blot analysis of cleaved Caspase-3, p21 and p-Akt from mammary gland extracts of PND21 rats exposed prepubertally via lactating dams treated with BPA and/or genistein, and SO. **(A)** Top panel depicts the Western blots of mammary gland proteins from SO controls (lanes 1, 5, 9, 13, 17 and 21), BPA (lanes 2, 6, 10, 14, 18 and 22), genistein (lanes 3, 7, 11, 15, 19 and 23) and BPA plus genistein (lanes 4, 8, 12, 16, 20 and 24) -treated groups with the appropriate antibodies shown on the left. For each treatment group, n = 6. **(B)** Densitometric values of Western blots from PND21 rats were reported as a percentage of controls ± SEM. *p ≤ 0.05 compared with controls; **p ≤ 0.05 compared with BPA; ***p ≤ 0.05 compared with genistein. No significant difference was detected for the house keeping protein GAPDH.

To determine the mammary gland’s molecular response to chemical exposure during the prepubertal period, we used western blot analysis to examine protein expression of signalling pathways linked to cell proliferation and apoptosis in the mammary glands of PND21 rats. We found that, as compared to control, prepubertal BPA treatment significantly increased p21 protein expression but had no effect on cleaved caspase-3 and p-Akt, while genistein treatment resulted in decreased cleaved caspase-3 and increased p-Akt protein expression (Figure 2).

Interestingly, combinational BPA + Gen treatment decreased cleaved caspase-3 and p21 compared to BPA treatment alone and decreased p-Akt protein compared to genistein treatment alone. Only the 19 kDa fragment of cleaved caspase-3 was detected, not the 17 kDa fragment at PND21. We also investigated Akt-1, Akt-2, Akt-3, cleaved caspase-9, p-Bad, PARP, PTEN, ER-α, ER-β, SRC-1, SRC-2 and SRC-3 protein expressions at PND21 but found no differences between treatment groups at this age (data not shown).

### Cellular proliferation and apoptosis in mammary glands of PND50 rats

**Figure 3:**
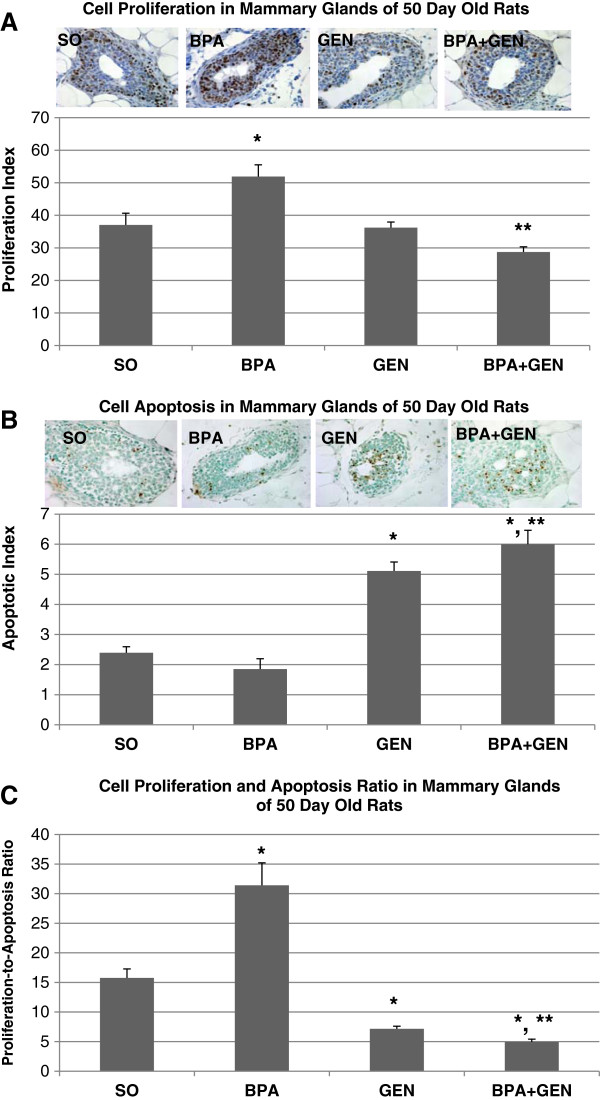
**Cell proliferation and apoptosis in PND50 rats.** Cell proliferation **(A)**, apoptosis **(B)**, and cell proliferation-to-apoptosis ratio **(C)** in mammary gland TEB epithelial cells of PND50 rats exposed prepubertally via lactating dams treated with BPA and/or genistein (n = 6 rat/treatment) from PND2 until PND20. Values represent mean ± SEM. *p ≤ 0.01 compared with controls; **p ≤ 0.001 compared with BPA.

Because Sprague Dawley rats are most susceptible to chemically-induced mammary cancer at PND50 [14], we also investigated cell proliferation and apoptosis at that age. Prepubertal BPA treatment significantly increased cell proliferation in mammary gland TEBs as compared to controls (p < 0.01) (Figure 3A). On the other hand, combinational BPA + Gen treatment significantly reduced cell proliferation when compared to BPA only treatment (p < 0.001). In regard to apoptosis, genistein treatment alone significantly increased cell apoptosis in the mammary glands of PND50 rats (p < 0.01) (Figure 3B). While prepubertal BPA only treatment had no significant effect on apoptosis in the mammary gland TEBs of PND50 rats, combinational BPA + Gen treatment significantly increased the index of apoptosis when compared to controls (p < 0.01) and BPA only (p < 0.001) treated rats.

When proliferation and apoptosis were taken together as an indicator of cellular turnover in mammary TEBs [12], prepubertal BPA treatment alone significantly increased cellular proliferation-to-apoptosis ratio compared with control treatment (p < 0.01) (Figure 3C). However, genistein exposure alone significantly decreased the cell proliferation to apoptosis ratio (p < 0.01). BPA + Gen combinational exposure also significantly decreased the cell proliferation to apoptosis ratio when compared with control (p < 0.01) and BPA treatment alone (p < 0.001), similar to the effects of genistein only treatment (Figure 3C).

### Protein expression in mammary glands of PND50 rats

**Figure 4:**
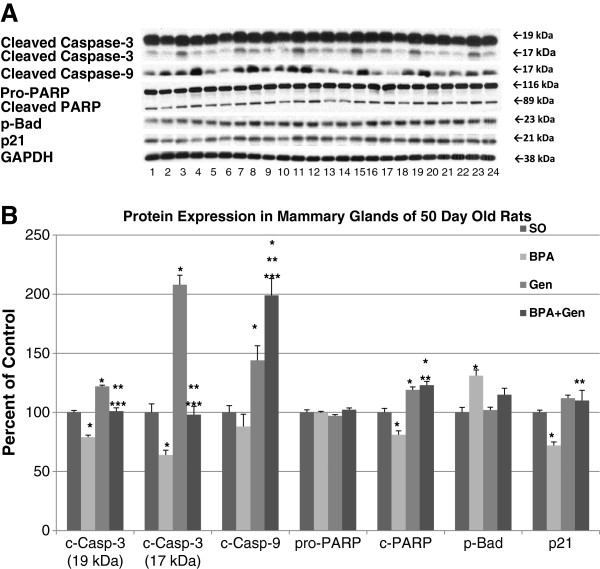
**Protein expressions in PND50 rats.** Western blot analysis of cleaved Caspase-3, cleaved Caspase-9, pro-PARP, cleaved PARP, p-Bad and p21 protein expression in mammary glands of PND50 rats exposed prepubertally via lactating dams treated with BPA or/and genistein and SO. **(A)** Top panel depicts the Western blots of mammary gland proteins from SO (lanes 1, 5, 9, 13, 17 and 21), BPA (lanes 2, 6, 10, 14, 18 and 22), genistein (lanes 3, 7, 11, 15, 19 and 23) and BPA plus genistein (lanes 4, 8, 12, 16, 20 and 24) -treated groups with the appropriate antibodies shown on the left. For each treatment group, n = 6. **(B)** Densitometric values of Western blots from PND50 rats were reported as a percentage of the controls ± SEM: *p ≤ 0.05 compared with controls; **p ≤ 0.05 compared with BPA; and ***p ≤ 0.01 compared with genistein. No significant difference was detected for the house keeping protein GAPDH.

**Figure 5:**
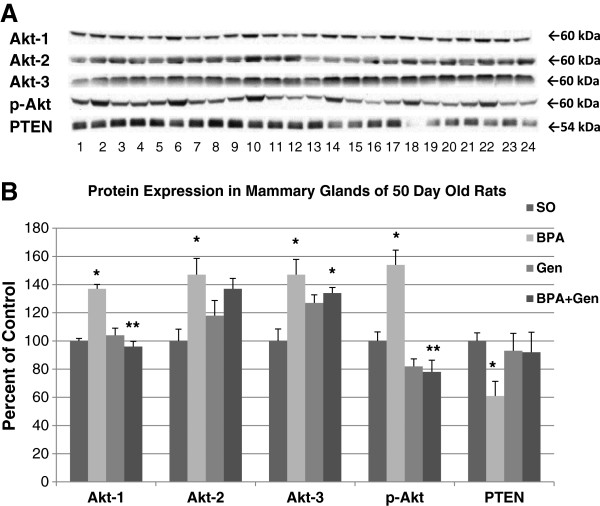
**Protein expressions in PND50 rats.** Protein expressions of Akt-1, Akt-2, Akt-3, p-Akt and PTEN from mammary gland extracts of PND50 exposed prepubertally via lactating dams treated with BPA or/and genistein, and SO. **(A)** Top panel depicts the Western blots of mammary gland proteins from SO (lanes 1, 5, 9, 13, 17 and 21), BPA (lanes 2, 6, 10, 14, 18 and 22), genistein (lanes 3, 7, 11, 15, 19 and 23) and BPA plus genistein (lanes 4, 8, 12, 16, 20 and 24) -treated groups with the appropriate antibodies shown on the left. For each treatment group, n = 6. **(B)** Densitometric values of Western blots from PND50 rats were reported as a percentage of the controls ± SEM: *p ≤ 0.05 compared with controls; **p ≤ 0.001 compared with BPA.

**Figure 6:**
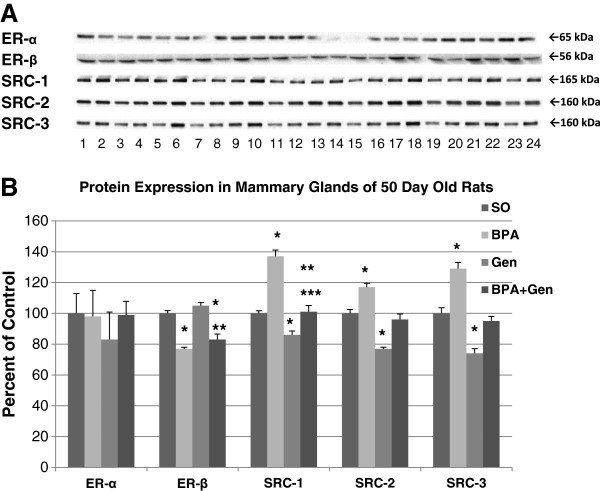
**Protein expressions in PND50 rats.** Protein expressions of ER-α, ER-β, SRC-1, SRC-2 and SRC-3 from mammary gland extracts of 50 day old rats exposed prepubertally via lactating dams treated with BPA or/and genistein, and SO. **(A)** Top panel depicts the Western blots of mammary gland proteins from SO (lanes 1, 5, 9, 13, 17 and 21), BPA (lanes 2, 6, 10, 14, 18 and 22), genistein (lanes 3, 7, 11, 15, 19 and 23) and BPA plus genistein (lanes 4, 8, 12, 16, 20 and 24) -treated groups with the appropriate antibodies shown on the left. For each treatment group, n = 6. **(B)** Densitometric values of Western blots from PND50 rats were reported as a percentage of the controls ± SEM: *p ≤ 0.05 compared with controls; **p ≤ 0.05 compared with genistein; ***p ≤ 0.0001 compared with BPA.

Because of the statistically significant alterations in cell proliferation and apoptosis in mature rats, and the reported susceptibility of PND50 rats being especially susceptible for mammary cancer [14], we measured the expression of several proteins commonly linked to cell cycle and apoptosis at PND50. In mammary glands of rats exposed prepubertally to BPA, we found that cleaved caspase-3, cleaved PARP and p21 were significantly down-regulated, while p-Bad was significantly increased compared to control animals (no effect on cleaved caspase-9) (Figure 4). On the other hand, prepubertal genistein treatment increased cleaved caspase-3, cleaved caspase-9, and cleaved PARP compared to control treated rats (no significant effect on p-Bad and p21). Interestingly, mammary glands of PND50 rats exposed prepubertally to combinational BPA + Gen had increased protein expressions of cleaved caspase-3, cleaved caspase-9, cleaved PARP and p21 compared to BPA only exposed rats. Cleaved caspase-9 and cleaved PARP were also up-regulated in animals exposed to combinational BPA + Gen treatment compared to controls. In regard to cleaved caspase-3, the 17 kDa and 19 kDa fragments were regulated similarly compared to controls. Inactive pro-PARP protein expression was not significantly altered between any treatment groups. Prepubertal BPA, but not genistein only, treatment significantly increased the expression of the Akt-1, -2, and -3 isoforms and p-Akt, and decreased the expression of PTEN compared to controls in PND50 rats (Figure 5). On the other hand, the mammary glands of combinational BPA + Gen treatment had significantly decreased Akt-1 and p-Atk protein expression compared to BPA only treatment.We assessed the expression of ER-α, ER-β and steroid receptor co-regulator (SRC) proteins known to play a role in estrogen signalling and breast cancer following prepubertal exposure to BPA or/and genistein. No significant differences were observed in ER-α expression. However, BPA and combinational BPA + Gen, but not genistein only treatment, reduced the expression of ER-β (Figure 6).

Also, we found all three members of the p160 family of SRC proteins, SRC-1, SRC-2 and SRC-3, were significantly up-regulated in response to prepubertal exposure to BPA, but down-regulated from prepubertal genistein exposure when compared with controls. Furthermore, the combinational exposure of BPA + Gen significantly decreased the SRC proteins when compared with BPA alone.

## Discussion

Cell turnover is a key process involved in mammary gland development and the pathogenesis of tumor formation; hence we investigated if early prepubertal exposure to BPA and genistein treatment, alone and in combination, exerted effects on cell proliferation and apoptosis. At PND21, rats prepubertally exposed to genistein only exhibited a 50% increase in cell proliferation in mammary TEBs (p < 0.01), a slight but not significant decrease in apoptosis, and a significant increase in cell turnover (2.6-fold). These effects are consistent with previous reports of prepubertal genistein treatment inducing rapid mammary gland growth that enhanced mammary gland maturation and cell differentiation (at PND50) and decreased susceptibility for chemically-induced mammary cancer in adult rats [8-10]. On the other hand, there was no significant change in cell proliferation or apoptosis following prepubertal BPA only exposure at PND21. However, the combined BPA + Gen treatments significantly increased cell proliferation and decreased apoptosis, resulting in a 3.6-fold increase in cell turnover at PND21. Since the results of combinational BPA + Gen treatment are most similar to the outcomes observed for genistein only treatment, we hypothesize that genistein is able to exert its effects on mammary gland maturation and differentiation even in the presence of BPA. While cell proliferation and apoptosis were measured in PND21 rats, it is important to point out that the observed effects at this age may be a consequence of the accumulative effects that occurred from the direct presence of BPA and/or genistein on days 2–21.

Subsequently, we investigated cell proliferation and apoptosis in PND50 rats exposed prepubertally to BPA and genistein, alone and in combination. This is 30 days after the last treatment, and a time point that, due to metabolism, animals are free of circulating genistein and BPA. Nevertheless, mammary TEBs of rats that were exposed prepubertally to BPA alone had increased cell proliferation and cell turnover by 40% and 99%, respectively. However, prepubertal genistein only treatment significantly increased cell apoptosis and decreased cell turnover (55%). While BPA did not alter apoptosis and genistein did not affect cell proliferation, both single treatments changed the ratio of cell proliferation-to-apoptosis. Furthermore, the combination of BPA + Gen exposure on the ratio of cell proliferation to apoptosis was similar to genistein only treatment (decreases), suggesting that prepubertal genistein action could overcome the action of prepubertal BPA treatment towards susceptibility for carcinogenesis.

Because of the differential effects of BPA and genistein on cell proliferation and apoptosis observed on PND50, we utilized western blot analysis to investigate proteins and the pathways targeted by these chemicals. In the mammary glands of rats exposed prepubertally to BPA only, cleaved caspase-3, cleaved PARP and p21 were significantly down-regulated while p-Bad was significantly increased compared to control treated animals, intimating decreased apoptosis. A failure to undergo apoptosis whereby DNA damaged cells are allowed to replicate is widely believed to be a key event in cancer formation and progression [15,16]. We believe the increase in mammary cancer that occurs in rats exposed prepubertally to BPA only and later to the DNA damaging carcinogen DMBA at PND50 falls in this category [9,11,12].

On the other hand, prepubertal genistein only treatment increased cleaved caspase-3, cleaved caspase-9 and cleaved PARP compared to the mammary glands of control treated rats. Therefore, differential regulation of cleaved caspase-3 and cleaved PARP was observed in comparing BPA and genistein treatments. The results of increased cleaved caspase-9 and cleaved PARP in genistein alone and BPA + Gen groups were consistent with our cell apoptosis data. PARP is a well-established substrate for caspase-3 [17], and cleaved PARP is considered to be a hallmark of apoptosis [18]. Western blot detection of PARP cleavage has been used extensively as an indicator of apoptosis [19].

The PI3K pathway is an intracellular signalling pathway important in the regulation of apoptosis and cancer development, being overactive when the tumor suppressor PTEN is faulty or deficient [20]. Also, playing a role in apoptosis are the Akt proteins, which promote growth factor-mediated cell survival both directly and indirectly. BAD is a pro-apoptotic protein of the Bcl-2 family. Akt could phosphorylate BAD on Ser136, which makes Bad dissociate from the Bcl-2/Bcl-X complex and lose the pro-apoptotic function [21]. In regard to the action of these proteins, we found that BPA only, but not genistein only, treatment significantly increased the expression of the Akt-1, 2, and 3 isoforms, p-Bad and p-Akt, but decreased on PTEN compared with control treatment, suggesting that BPA could inhibit cell apoptosis by regulation of PTEN, Akt pathway, and p-Bad in rat mammary glands. On the other hand, Akt-1 and p-Akt were significantly decreased in the mammary glands of rats exposed to combinational BPA + Gen treatment compared to BPA only treatment, pointing to the protective effect of genistein over BPA for apoptosis over cell proliferation.

Steroid hormones can exert their actions by signalling through intracellular steroid hormone receptors. Regulation of steroid receptor action, including ER-α and ER-β, is complex due to a number of co-regulators, including steroid receptor co-activators, that can determine signalling specificity and intensity, resulting in pleiotropic biological effects [22,23]. But, it is not only steroid molecules that act via these mechanisms. We have previously reported that prepubertal BPA exposure resulted in up-regulated SRCs1-3, without altering ER-α in the mammary glands of 50 day old rats [9]. Here, we confirm those effects on the SRCs, plus demonstrate that prepubertal BPA exposure results in reduced expression of ER-β. ERβ has been shown to inhibit breast cancer cell growth by reducing cell proliferation and has been termed a tumor suppressor [24-26]. Decreased ER-β has been associated with proliferative preinvasive mammary tumors [27]. So, the decreased expression of ER-β by BPA suggests increased susceptibility for chemically-induced mammary carcinogenesis in adult rats. Furthermore, increased expression of the SRCs could have a dual effect, one to enhance ERβ action as a suppressor of mammary cancer and the other to stimulate “normal” levels of ER-α to enhance tumor-promoting effects. In contrast, prepubertal genistein exposure resulted in down-regulated expression of the SRCs, leading to the opposite effect of BPA for carcinogenesis via SRC regulation, i.e. mammary cancer prevention. Consistent with the actions of genistein only, the SRCs were down-regulated in mammary glands of rats exposed to BPA + Gen, suggesting that genistein may reduce the potential of BPA’s carcinogenic effect towards mammary cancer development.

In summary, prepubertal BPA exposure had little direct effect on cell proliferation and apoptosis and altered protein expression of only one out of 15 proteins investigated at PND21. Nevertheless, in mature rats (30 days later) cell proliferation and the ratio of cell proliferation to apoptosis were significantly increased. Associated with this at PND50 are differential expressions of 15 proteins whose alterations are consistent with increased cell proliferation and reduced apoptosis. These data are supportive of prior reports of rats exposed in a similar manner to BPA prepubertally and at PND50 to DMBA developing more mammary tumors [11,12]. We hypothesize that DNA damaged cells from DMBA exposure at PND50 that did not undergo apoptosis were allowed to undergo long-term rapid cell proliferation to promote mammary tumor development. Our collaborative epigenetic studies have shown that mammary epithelial cells from PND50 rats exposed prepubertally to BPA revealed concurrent repression in 10 genes homologous to the 16p11.2 loci [28]. These findings suggest that this conserved cluster is susceptible to BPA-mediated repression in rat mammary epithelial cells and may lead to permanent silencing of the 16p11.2 loci, which in turn can effect cell cycle regulation. Also, there were another 75 rat genes found to be down-regulated.

Interestingly enough, the mechanism for genistein chemoprevention is linked to increased cell proliferation that takes place prepubertally. As evident here, prepubertal genistein exposure results in increased cell proliferation in epithelial cells of mammary TEBs in prepubertal rats. This is correlated with increased mammary gland maturation and differentiation of TEBs to lobules [8,10]. While TEBs are the terminal ductal structures most susceptible to carcinogenesis in the rat mammary gland, lobules are the terminal ductal structures least susceptible to carcinogenesis [13]. Furthermore, prepubertal genistein exposure results in increased apoptosis at PND50, which could reduce the number of DNA damaged cells.

Combinational exposures are considered by many to be rather complex to be studied, especially in vivo. For this reason, we selected two chemicals whose properties we have previously studied and have shown to have opposing outcomes as detailed above. While genistein has been shown to elicit estrogen-like properties, including stimulating cell proliferation at PND21 and promoting mammary gland maturation and cell differentiation, its toxicological properties are questionable or minimal, especially when administered orally. Several laboratory and epidemiological studies have demonstrated genistein and its natural and most abundant source, soy, to suppress mammary cancer [8-10,29]. In this study we found that combinational BPA + Gen exposure, via the mothers’ milk, from PND2-21 resulted in increased cell proliferation and decreased apoptosis when investigated at PND21. Furthermore, three proteins presumed to be associated with these actions were down-regulated at PND21. At PND50, we found that combinational BPA + Gen exposure resulted in significantly decreased cell proliferation and increased apoptosis. These results were very similar to the actions of prepubertal genistein only exposure. Also, nine proteins associated with cell proliferation and apoptosis were differentially regulated in a manner consistent with suppressing cell proliferation and increased potential for apoptosis at PND50. We interpreted this to mean that genistein could protect against BPA predisposition for mammary carcinogenesis. Our data strongly suggest that the mechanism behind these cellular responses are centred on differential protein expressions of caspases, PARP, Bad, p21, Akts, PTEN, ER-β and SRCs 1–3 in the rat mammary gland. Studies are under way to determine if these cellular actions supported by appropriate changes in long-term protein expressions can be explained by epigenetic mechanisms and translated into genistein suppressing BPA predisposition for mammary cancer.

## Conclusions

Genistein administered to rats during the prepubertal period stimulates mammary cell proliferation at PND21, but not at PND50, while apoptosis is increased at the latter age. The increase in cell proliferation during early postnatal mammary gland development has previously been shown to promote differentiation of the mammary terminal ductal structures and protect against chemically-induced cancer in rats [8,10]. Of added importance is that increased rate of apoptosis could also contribute to chemoprevention by killing DNA damaged cells. On the other hand, prepubertal BPA exposure increased cell proliferation in mammary glands of PND50 rats, events that have previously been associated with BPA predisposing for chemically-induced mammary cancer at day 50 [11]. Importantly, combinational prepubertal exposure to BPA + Gen resulted in increased cell proliferation in PND21 rats, and decreased cell proliferation and increased apoptosis in PND50 rats. The altered mechanisms behind these cellular responses can be explained on the basis of differential protein expressions in the rat mammary gland. It appears that early exposure to genistein can program for a “biochemical blueprint” in mammary terminal ductal structures that can suppress mammary cancer susceptibility [8-10,30].

## Competing interests

The authors declare that they have no conflicts of interest.

## Authors’ contributions

CAL conceived and designed the experiments. JW and SJ performed the experiments and analyzed the data. JW, SJ and CAL wrote the paper. All authors read and approved the final version of the manuscript.

## Pre-publication history

The pre-publication history for this paper can be accessed here:

http://www.biomedcentral.com/1471-2407/14/379/prepub
